# Peritumoral tertiary lymphoid structure and tumor stroma percentage predict the prognosis of patients with non-metastatic colorectal cancer

**DOI:** 10.3389/fimmu.2022.962056

**Published:** 2022-09-16

**Authors:** Qianyu Wang, Xiaofei Shen, Ran An, Junchao Bai, Junhua Dong, Huiyun Cai, Hongyan Zhu, Wentao Zhong, Wenliang Chen, Aijun Liu, Junfeng Du

**Affiliations:** ^1^ The 2nd School of Clinical Medicine, Shanxi Medical University, Taiyuan, China; ^2^ Department of General Surgery, Affiliated Drum Tower Hospital of Nanjing University Medical School, Nanjing, China; ^3^ Department of Pathology, The 7th Medical Center, Chinese PLA General Hospital, Beijing, China; ^4^ Department of General Surgery, The 7th Medical Center, Chinese People's Liberation Army (PLA) General Hospital, Beijing, China; ^5^ The 2nd School of Clinical Medicine, Southern Medical University, Guangzhou, China; ^6^ Department of General Surgery, The 2nd Affiliated Hospital of Shanxi Medical University, Taiyuan, China; ^7^ Medical Department of General Surgery, The 1st Medical Center, Chinese People's Liberation Army (PLA) General Hospital, Beijing, China

**Keywords:** colorectal cancer, tumor immune microenvironment, tertiary lymphoid structures (TLS), tumor stroma percentage, nomogram, prognosis

## Abstract

**Background:**

Tertiary lymphoid structures (TLSs) are crucial in promoting and maintaining positive anti-tumor immune responses. The tumor stroma has a powerful immunosuppressive function that could exclude tumor-infiltrating lymphocytes from the tumor beds and lead to a “cold” phenotype. TLSs and tumor stroma percentage (TSP) are significantly associated with the prognosis of patients with certain cancers. However, the exact roles of TLSs and TSP and their intrinsic relationship are still largely unknown in colorectal cancer (CRC).

**Methods:**

TLSs and TSP were assessed using hematoxylin-eosin (H&E) and/or immunohistochemistry (IHC) staining from 114 CRC patients in the training set and 60 CRC patients in the external validation set. The correlation between TILs, TLS and clinicopathological characteristics and their prognostic values were assessed. Finally, we plotted a Nomogram including the TLS, TSP and tumor-node-metastasis (TNM) stage to predict the probability of recurrence-free survival (RFS) at 2- and 5-years in non-metastatic colorectal cancer (nmCRC) patients.

**Results:**

Peritumoral TLS (P-TLS), intratumoral TLS (In-TLS) and high TSP (H-TSP, >50%) were present in 99.1%, 26.3% and 41.2% patients, respectively. H-TSP tumor tends to be associated with lower P-TLS density (*P* =0.0205). The low P-TLS density (< 0.098/mm^2^) was significantly associated with reduced RFS (HR=6.597 95% CI: 2.882-15.103, *P <*0.001) and reduced overall survival (OS) (HR=6.628 95% CI: 2.893-15.183, *P* < 0.001) of nmCRC patients. In-TLS was not of significance in evaluating the clinical outcomes of nmCRC patients. H-TSP was significantly associated with reduced RFS (HR=0.126 95% CI: 0.048-0.333, *P <*0.001) and reduced OS (HR=0.125 95% CI: 0.047-0.332, *P <*0.001) of nmCRC patients. The 5-year RFS of the high P-TLS, low-TLS, H-TSP, and L-TSP groups were 89.7%, 47.2%, 53.2%, and 92.5%, respectively. The P-TLS density, TSP and TNM stage were independent prognosis factors of nmCRC patients. The Nomogram, including the P-TLS density, TSP and TNM stage, outperformed the TNM stage.

**Conclusions:**

High P-TLS density and low TSP (L-TSP) were independent and favorable prognostic factors of nmCRC patients, which might provide new directions for targeted therapy in the CRC tumor microenvironment, especially the tumor immune microenvironment.

## Introduction

The latest data show that colorectal cancer (CRC) is the third most common and second most deadly malignancy worldwide (1). The host immune system is essential in tumor initiation and progression and is recognized as a good prognostic factor of CRC patients (2). Tertiary lymphoid structures (TLSs) are organized aggregates of immune cells that develop in non-lymphoid tissues at sites of chronic inflammation such as in autoimmune disease, chronic infection, and cancer (3). TLSs display a pronounced anatomical and functional resemblance to secondary lymphoid organs (lymph nodes and Peyer’s patches) but lack the surrounding capsule (4). This absence of encapsulation may permit direct access of their cellular components to the surrounding tissue and might be necessary to recruit lymphocytes and to maintain an optimal immune microenvironment (3, 5, 6). More and more evidence shows that high TLS density is associated with a good prognosis for lung cancer (7, 8), oral cancer (9), pancreatic cancer (10), breast cancer (11, 12), and colorectal cancer (13, 14) and this prognostic value is independent of tumor-node-metastasis (TNM) stage, such aslung cancer (7), pancreatic cancer (10) and CRC (13). However, other studies suggest that TLS density has no prognostic value in CRC patients, especially in stage III (6, 15). Therefore, the exact role of TLS requires further validation in CRC patients.

As an indispensable element of the tumor microenvironment, tumor stroma is essential for tumor initiation, progression, metastasis, and treatment resistance (16, 17). Recent studies have identified the strong immunosuppressive activity of tumor stroma as the primary mechanism by which tumor stroma can promote tumor progression and confer resistance to immunotherapy. In the context of tumor immunity, the most prominent effect of tumor stroma is to exclude tumor-infiltrating T cells from the tumor beds, resulting in a “cold” phenotype (18). Specifically, stroma cells, such as macrophages and cancer-associated fibroblasts, could reduce T cell motility by secreting various immunosuppressive cell cytokines, leading to an “immunoexclusion” phenotype (19, 20). In addition, the dense stromal structure presents unique challenges for T cell infiltration (21–23). High tumor stroma percentage (TSP) is associated with poor prognosis and suppression of immune microenvironment (24).

In this study, TLS and TSP were evaluated in two independent sets. The intrinsic relationship between TLS and TSP, and their prognostic value were assessed. Finally, the Nomogram, including the P-TLS density, TSP and TNM stage, could guide clinicians to more accurately identify non-metastatic colorectal cancer (nmCRC) patients with a poor prognosis.

## Materials and methods

### Patient population

This study included two independent sets. The training set included 114 nmCRC patients who underwent elective laparoscopic surgery, histologically confirmed adenocarcinoma and harvested at least 12 lymph nodes at the 7th Medical Center of Chinese PLA General Hospital between January 2015 and June 2016. This study excluded the patients who received neoadjuvant therapy (including chemotherapy, radiotherapy and chemoradiotherapy), underwent emergency resection, died within 30 days after surgery or with dual or multiple primary tumors. Based on the same inclusion and exclusion criteria, the external validation set included 60 nmCRC patients at the Affiliated Drum Tower Hospital of Nanjing University Medical School between January 2016 to January 2017. The local ethics committee approved this study protocol.

### Clinicopathological characteristics

Clinicopathological characteristics, including vascular and perineural invasion and mismatch repair (MMR) status, were obtained from pathology reports. The TNM stage was re-evaluated by the eighth edition TNM staging system. The patients’ demographic information included age, sex and tumor location. The tumor location was classified as left, right colon, and rectum. All patients were routinely followed up for five years after CRC resection. Censored Data was defined as patients alive at the last follow-up and those who died from non-CRC causes.

All available hematoxylin and eosin (H&E)-stained sections were reviewed and the representative sections were selected. These representative sections should include the tumor tissue and normal tissue surrounding the tumor tissue. The corresponding tissues were subsequently resected into 4-μm thick slices. All the representative H&E-stained sections were scanned using MoticEasyScan Infinity and analyzed using Motic DSAssistant.

### Assessment of mismatch-repair status and vascular invasion

Primary antibodies to the mismatch repair (MMR) proteins MLH1, PMS2, MSH2 and MSH6 were used for MSI analysis. All tumor cells lacked nuclear labeling, whereas normal epithelial and stromal cells maintained their staining, indicating loss of the corresponding MMR protein. If any of the four MMR proteins were preserved, the tumor was considered mismatch repair-proficient (pMMR). Tumors that lose the expression of any of the four proteins were considered mismatch repair-deficient (dMMR)

D2-40 and CD31 are labeled as lymphatic and blood vessels, respectively. Vascular invasion was defined as invasion of cancer cells into the walls of lymphatic or blood vessels, or the detection of tumor thrombi in the lumen.

### Assessment of the TLS

**Figure 1:**
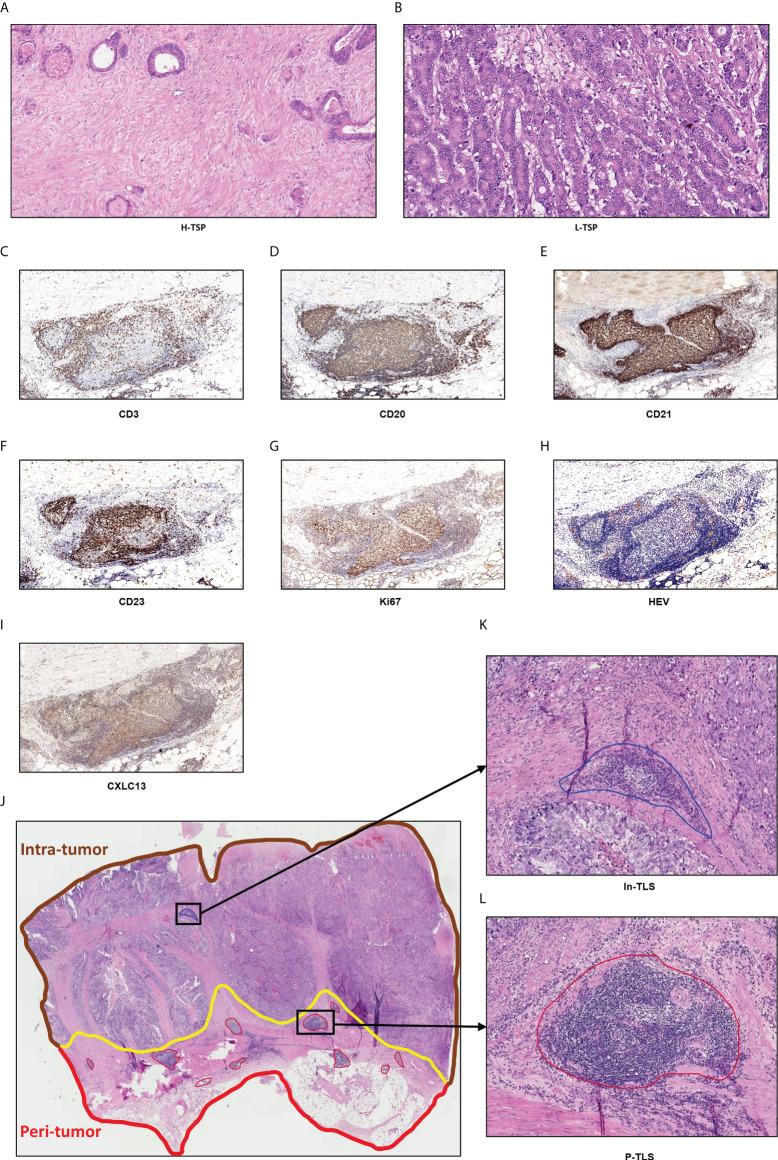
Hematoxylin and eosin-stained (H&E) sections of tumor stroma percentage and TLS. **(A)** High TSP (>50%). **(B)** Low TSP (≤50%). Germinal centers (GCs) were confirmed to contain strictly confined regions of CD21 follicular dendritic cells in a field of larger CD23 and Ki67 B cells (CD20) surrounded by PNAd vessels **(C–I)**. Peri-tumor region was surrounded in red while intra-tumor region was surrounded in brown **(J)**. In-TLS **(K)** and P-TLS **(L)** are highlighted in blue and red, respectively.

TLSs were defined as dense aggregates of B cells (CD20) with an adjacent T-cell zone (CD3) lacking the surrounding capsule. TLS maturation stages were analyzed in all patients by the detection of FDCs (CD21), germinal center (GC), CXCL13 and peripheral node addressin (PNAd) -positive high endothelial venules (HEV). Early (E)-TLS contains: primarily clusters of T and B cells without FDC network (CD21) and GC; Primary follicle-like (PFL)-TLS contains: clusters with an FDC network without GC; Secondary follicle-like (SFL)-TLS contains: lymphocyte clusters containing distinct yet adjacent T- (CD3) and B cell (CD20) zones, expression of the chemokines CXCL13 and GC reactions in the B cell follicle. GCs were confirmed to contain strictly confined regions of CD21 follicular dendritic cells in a field of larger CD23 and Ki67 B cells (CD20) surrounded by PNAd+ vessels (**Figures 1C-I**) (25).

As shown in **Figures 1J-L**, TLS was divided into peritumoral TLS (P-TLS) and intratumoral TLS (In-TLS) based on their location to tumor invasive margins. The number of TLS per square millimeter in a 7-mm area around the tumor was defined as the P-TLS density. The number of TLS per square millimeter in the tumor area was defined as the intratumoral In-TLS density.

Immunohistochemical (IHC) staining was performed on successive sections to further accurately assess TLS-cellular components, which include immunoactive cells such as CD8+ T cells, CD45RO+ lymphocytes cells, CD4+ T cells, CD20+ B cells, dendritic cells (DCs) and natural killer cells (NKs), and immunosuppressive cells such as regulatory T cells (Tregs), tumor-associated macrophages (TAMs) and tumor-associated neutrophils (TANs). Percentages of each TLS-cellular component were defined as the ratio of the number of positive cells to the number of nucleated cells in a TLS. According to the location of the TLS, the mean percentages of each TLS-cellular components of all P-TLS and In-TLS were then calculated as the representative value for one patient.

### Immunohistochemistry

The slides were deparaffinized, rehydrated and then incubated at room temperature with 3% H_2_O_2_ for 10 min. Heat-mediated antigen repair was performed using antigen repair solution citrate buffer (10 mM pH 6). The slides were sealed with 5% bovine serum albumin (BSA) at room temperature for 30 min and then incubated with primary antibody at 4°C overnight. Corresponding primary antibody information is shown in **Supplementary Table 1**. The next day, the sections were incubated with secondary antibody for 1 hour and diaminobenzidine (DAB) for 3 min, followed by hematoxylin staining. The sections were then dehydrated and sealed in neutral resin. The sections were scanned using Leica CS2 and analyzed using Aperio ImageScope.

### Assessment of the TSP

TSP was identified using the corresponding H&E-stained sections by two independent observers. The most invasive tumor field was selected at high magnification (×100) and tumor cells would have to be present on all field boundaries. TSP was defined as the percentage of tumor stroma in the field. In this study, TSP >50% was considered as high TSP (H-TSP) and TSP ≤50% as Low TSP (L-TSP) (**Figures 1A, B**), as described by Park (26). When there were different opinions, discussions were held and the senior doctor made the final decision.

### Statistical analysis

Statistical analyses were performed with R (version 4.1.3), GraphPad Prism (v.7) and SPSS version 25 (IBM, Armonk, NY, USA). Mann-Whitney tests were used to compare continuous variables. Chi-square or Fisher’s exact tests were used to compare categorical variables. Recurrence-free survival (RFS) and overall survival (OS) were compared using the Kaplan-Meier and log-rank survival analysis. Hazard ratios (HR) and 95% confidence intervals (CI) were calculated using the Cox regression analysis. The variables included in multivariate analysis were significantly (*p <*0.05) associated with prognosis in univariate analysis. All statistical analyses were based on two-sided analysis and a *p* value of <0.05 was considered as statistically significant difference.

Based on multivariate Cox-regression analyses, the Nomogram, including the P-TLS density, TSP and TNM stage, was constructed to predict the probability of RFS at 2- and 5-years in nmCRC patients. The discriminative ability of the Nomogram was examined by the concordance index (C-index), with 0.5 being none discriminating ability and 1.0 being a perfectly discriminating ability. Calibration curves were utilized to evaluate the link between actual and predicted clinical outcomes.

## Results

### Demographics and clinical characteristics

We enrolled 114 and 60 nmCRC patients in the training and external validation set, respectively, and the demographics and clinical characteristics were summarized in **Table 1**. 47 (41.2%) patients were lymph node-positive. 23 (20.2%) and 19 (16.7%) patients were perineural and vascular invasion, respectively. 22 (19.3%), 45 (39.5%) and 47 (41.2%) cases were TNM stage I, II and III, respectively. Mismatch repair-deficient (dMMR) was observed in 17 (14.9%) cases. In the training set, the recurrence rates for 1-, 2-, and 5-years were 3.5%, 12.3% and 23.7%, respectively; and the survival rates for 1-, 2-, and 5-years were 99.1%, 89.5% and 76.3%, respectively.

**Table 1:** Demographics and clinical characteristics of nmCRC patients.

Characteristics	Training set n (%)	External validation set n (%)	P value
**Sex**			0.6280
Male	65 (57.0)	37 (61.7)	
Female	49 (43.0)	23 (38.3)	
**Age(years)**			0.0774
>60	41 (36.0)	30 (50.0)	
≤60	73 (64.0)	30 (50.0)	
**Location**			0.6240
Rectum	75 (65.8)	35 (58.3)	
Left colon	19 (16.7)	12 (20.0)	
Right colon	20 (17.5)	13 (21.7)	
**Lymph node-positive**			>0.9999
YES	47 (41.2)	25 (41.7)	
NO	67 (58.8)	35 (58.3)	
**Perineural invasion**			0.8425
YES	23 (20.2)	11 (18.3)	
NO	91 (79.8)	49 (81.7)	
**Vascular invasion**			0.6774
YES	19 (16.7)	12 (20.0)	
NO	95 (83.3)	48 (80.0)	
**T stage**			0.8991
T1	7 (6.1)	5 (8.3)	
T2	15 (13.2)	7 (11.7)	
T3	83 (72.8)	42 (70.0)	
T4	9 (7.9)	6 (10.0)	
**N stage**			0.7808
N0	67 (58.8)	35 (58.3)	
N1	30 (26.3)	18 (30.0)	
N2	17 (14.9)	7 (11.7)	
TNM stage*			0.9063
I	22 (19.3)	10 (16.7)	
II	45 (39.5)	25 (41.7)	
III	47 (41.2)	25 (41.7)	
**Tumor grade**			0.5119
G1	27 (23.7)	11 (18.3)	
G2	74 (64.9)	39 (65.0)	
G3	13 (11.4)	10 (16.7)	
**MMR**			0.8247
dMMR	17 (14.9)	8 (13.3)	
pMMR	97 (85.1)	52 (86.7)	
**TSP**			>0.9999
H-TSP	47 (41.2)	24 (40.0)	
L-TSP	67 (58.8)	36 (60.0)	
**P-TLS density (median)**	0.1535	0.1555	0.7266
**P-TLSmaturation stages**			0.5476
E-TLS	25 (22.1)	9 (15.3)	
PFL-TLS	51 (45.1)	28 (47.5)	
SFL-TLS	37 (32.7)	22 (37.3)	

nmCRC, non-metastatic colorectal cancer; MMR, mismatch repair; P-TLS, peritumoral tertiary lymphoid structure; E-TLS, early -TLS; TSP, PFL-TLS, primary follicle-like -TLS; SFL-TLS, secondary follicle-like -TLS; TSP, tumor stroma percentage. *The 8th AJCC TNM staging system

### Characteristics of TLS

**Figure 2:**
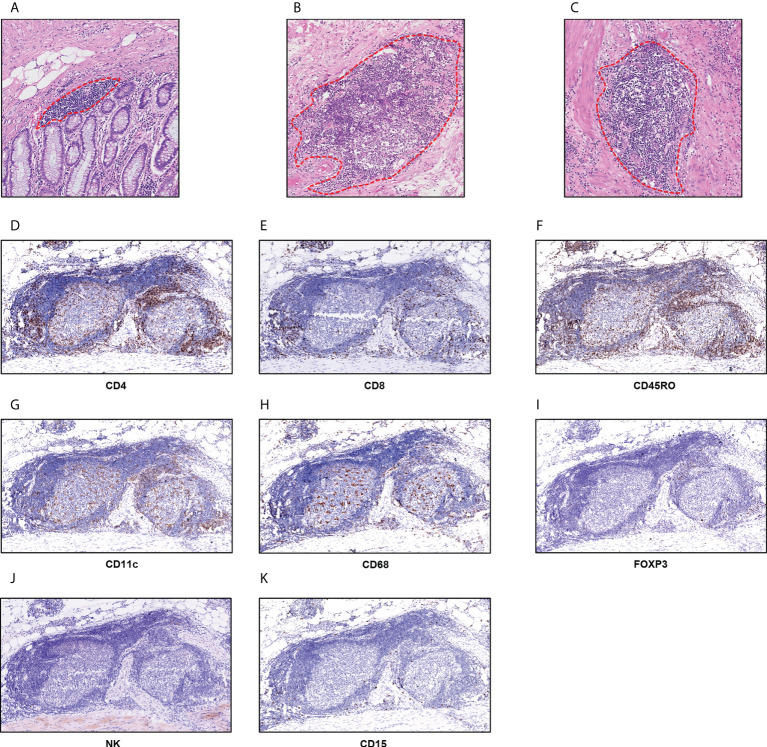
TLS in nmCRC. **(A)** The mucosal TLSs were almost squished and elongated or teardrop-shaped; **(B, C)** the submucosal and basal lamina propria TLSs were usually oval-shape; **(D)** CD4+ T cells; **(E)** CD8+ T cells; **(F)** CD45RO+ lymphocytes; **(G)** CD11c+DC cells; **(H)** CD68+ TAMs; **(I)** FOXP3+ Tregs; **(J)** NCR1+ NK cells; **(K)** CD15+ TANs.

In the training set, P-TLS and In-TLS were observed in 99.1% and 26.3% cases, respectively; and in the external validation set, P-TLS and In-TLS were observed in 100% and 28.3% cases, respectively. We found that the morphology and size of TLS were varied. The mucosal TLS was almost squished and elongated or teardrop-shaped, and the submucosal and basal lamina propria TLSs were usually oval-shaped (**Figures 2A-C**). The TLS-cellular components are shown in **Figures 2D-K**. B cells covered the whole TLS and DCs and TAMs were mainly located in the follicle region. In contrast, CD4+ T cells, CD8+ T cells, and CD45RO+ lymphocytes were mainly located in the T cell region and some dispersed to the center of the follicle. Tregs were sporadic. NK cells and TANs were rare in TLS.

**Figure 3:**
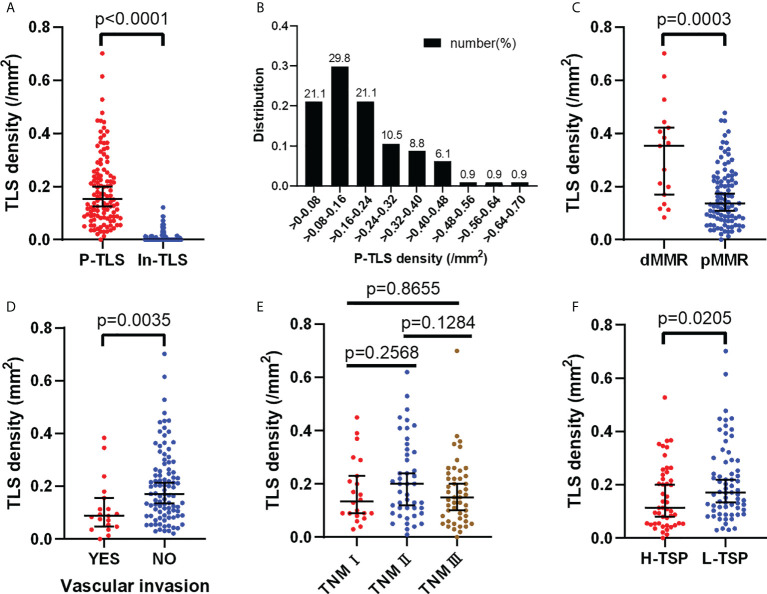
Distribution of P-TLS density and its relationship with clinical features in the training set. **(A)** Relationship between P-TLS density and In-TLS density; **(B)** Distribution of P-TLS density; **(C–F)** Relationship between P-TLS density and MMR/Vascular invasion/TNM stage/TSP.

P-TLS density is significantly higher than In-TLS density (*P <*0.0001, **Figure 3A**). The distribution of P-TLS density is shown in **Figure 3B**. Most nmCRC patients had P-TLS densities mainly concentrated in the 0-0.24/mm^2^ range, with a minority of nmCRC patients without or with higher P-TLS density (>0.24/mm2). We assessed the association between P-TLS density and clinicopathological characteristics in the training set (**Table 2**). Higher P-TLS density was observed in pMMR (Median: 0.3530 vs 0.1370, *P* =0.0003, **Figure 3C**) and vascular invasion-negative tumors (Median: 0.1710 vs 0.0880, *P* =0.0035, **Figure 3D**). P-TLS density was not significantly associated with TNM stage (**Figure 3E**). In addition, P-TLS density was not associated with other clinical features such as sex, age, tumor location, perineural invasion and tumor grade (**Table 2**). Similar results were obtained in the external validation set (**Supplement Table 2**; **Supplementary Figures 1A-F**).

**Table 2:** Relationship of TSP and TLS with clinicopathological characteristics of nmCRC patients in the training set.

Characteristics	Training set (n=114) n (%)				
	P-TLS density (median)	P value	H-TSP	L-TSP	P value
**Sex**		0.6679			0.7030
Male	0.1660 (0.0815, 0.2745)		28 (59.6)	37 (55.2)	
Female	0.1340 (0.0905, 0.2410)		19 (40.4)	30 (44.8)	
**Age**		0.1484			0.6953
>60	0.1340 (0.0710, 0.2380)		18 (38.3)	23 (34.3)	
≤60	0.1860 (0.0880, 0.2605)		29 (61.7)	44 (65.7)	
**Location**		0.8294			0.6595
Rectum	0.1370 (0.0810, 0.2480)		30 (63.8)	45 (67.2)	
Left colon	0.1630 (0.1010, 0.3320)		7 (14.9)	12 (17.9)	
Right colon	0.1680 (0.0980, 0.2480)		10 (21.3)	10 (14.9)	
**Lymph node-positive**		0.2305			**0.0002**
YES	0.1470 (0.0710, 0.2250)		29 (61.7)	18 (26.9)	
NO	0.1640 (0.0880, 0.3010)		18 (38.3)	49 (73.1)	
**Perineural invasion**		0.0947			0.4868
YES	0.1170 (0.0710, 0.1860)		11 (23.4)	12 (17.9)	
NO	0.1660 (0.0880, 0.2640)		36 (76.6)	55 (82.1)	
**Vascular invasion**		**0.0035**			**0.0022**
YES	0.0880 (0.0470, 0.1560)		14 (29.8)	5 (7.5)	
NO	0.1710 (0.0900, 0.2640)		33 (70.2)	62 (92.5)	
**T stage**		0.2449			**0.0384**
T1	0.1420 (0.0700, 0.3010)		1 (2.1)	6 (9.0)	
T2	0.1260 (0.0870, 0.2310)		4 (8.5)	11 (16.4)	
T3	0.1700 (0.0950, 0.2640)		35 (74.5)	48 (71.6)	
T4	0.0880 (0.0450, 0.1795)		7 (14.9)	2 (3.0)	
**N stage**		0.4319			**0.0009**
N0	0.1640 (0.0880, 0.3010)		18 (38.3)	49 (73.1)	
N1	0.1420 (0.0673, 0.2308)		18 (38.3)	12 (17.9)	
N2	0.1510 (0.0650, 0.2205)		11 (23.4)	6 (9.0)	
TNM stage*		0.2571			**0.0009**
I	0.1340 (0.0853, 0.2465)		5 (10.6)	17 (25.4)	
II	0.1990 (0.0970, 0.3270)		13 (27.7)	32 (47.8)	
III	0.1470 (0.0710, 0.2250)		29 (61.7)	18 (26.9)	
**Tumor grade**		0.2099			0.6992
G1	0.1250 (0.0710, 0.2140)		12 (25.5)	15 (22.4)	
G2	0.1465 (0.0870, 0.2693)		31 (66.0)	43 (64.2)	
G3	0.2030 (0.1340, 0.3185)		4 (8.5)	9 (13.4)	
**MMR**		**0.0003**			0.7902
dMMR	0.3530 (0.1520, 0.4325)		6 (12.8)	11 (16.4)	
pMMR	0.1370 (0.0780, 0.2320)		41 (87.2)	56 (83.6)	
**TSP**		**0.0205**	-	-	-
H-TSP	0.1130 (0.0540, 0.2380)		-	-	
L-TSP	0.1710 (0.1060, 0.2910)		-	-	
**Presence of In-TLS**		-			0.1953
Yes	-		9 (19.1)	21 (31.3)	
No	-		38 (80.9)	46 (68.7)	
**In-TLS density**	-	-	0.0064	0.0122	0.1568

P-TLS, peritumoral tertiary lymphoid structure; In-TLS, intratumoral TLS; TSP, tumor stroma percentage; H-TSP, high TSP; L-TSP, low TSP; nmCRC, non-metastatic colorectal cancer; MMR, mismatch repair. *The 8th AJCC TNM staging system. The bold valuse was considered as statistically significant difference.

### Association of TLS with Survival of nmCRC Patients

**Figure 4:**
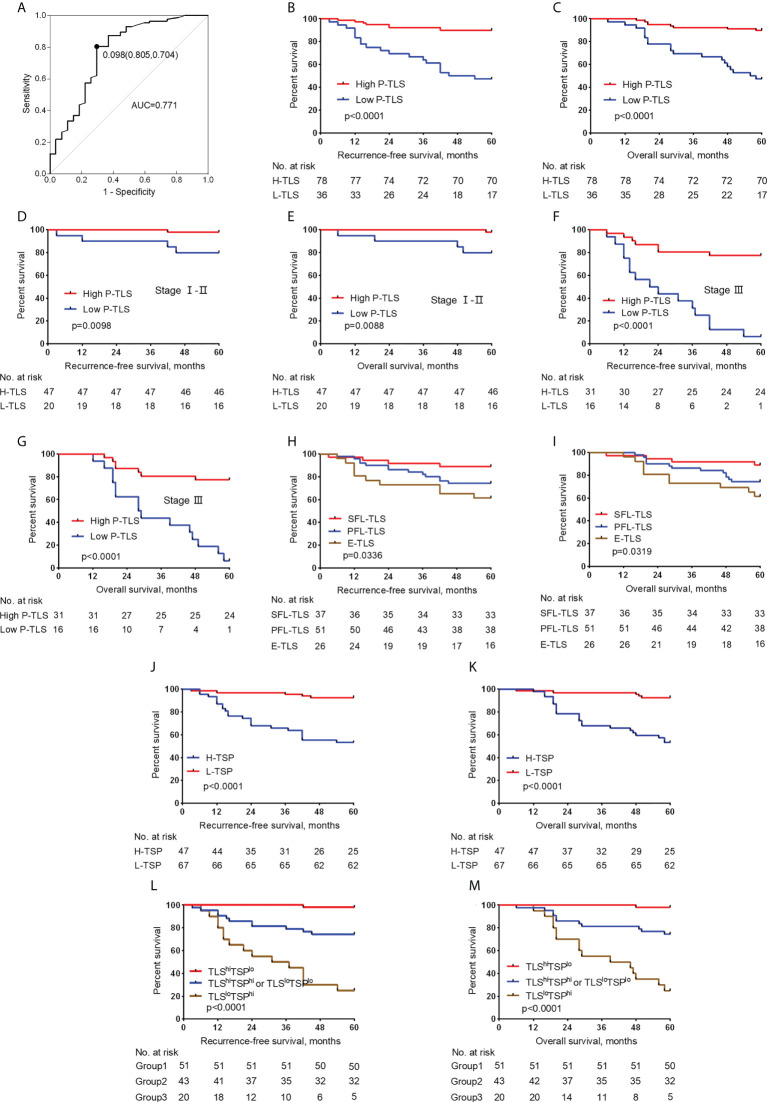
Relationship between the P-TLS and TSP and prognosis of nmCRC in the training set. **(A)** ROC curve of P-TLS density and patient prognosis. The optimum cut-off is 0.098, and when using this point for segmentation, the sensitivity is 0.805 and the specificity is 0.704. Association of the P-TLS density with RFS **(B)** and OS **(C)**, and TNM I-II **(D, E)** and III **(F, G)** CRC patients. Association of the P-TLS maturation stage with RFS **(H)** and OS **(I)**. Association of the TSP with RFS **(J)** and OS **(K)**. Kaplan-Meier survival analyses for RFS **(L)** and OS **(M)** were performed according to group 1, group 2, and group 3. Group 1: TLS^hi^TSP^lo^ group, group 2: TLS^hi^TSP^hi^ or TLS^lo^TSP^lo^ group, and group 3: the TLS^lo^TSP^hi^ group.

We evaluated the potential role of TLS in nmCRC patients. Since TLS in different locations might present different functions (27), we assessed the predictive effects of In-TLS and P-TLS, respectively (**Table 3**). In the training set, the optimum cut-off of P-TLS density was assessed based on ROC curves for 5-year RFS and then patients were divided into high P-TLS density and low P-TLS density groups. The optimum cut-off for P-TLS density was 0.098, with an area under the ROC curve (AUC) was 0.771 (95% CI: 0.659-0.884, **Figure 4A**). We found that low P-TLS density was significantly related to reduced RFS (HR=6.597 95% CI: 2.882-15.103, *P <*0.001, **Figure 4B**) and reduced OS (HR=6.628 95% CI: 2.893-15.183, *P <*0.001, **Figure 4C**) of nmCRC patients and this prognostic value independent of TNM stage (**Figures 4D-G**). We applied the optimum cut-off of P-TLS density to the external validation set and obtained similar results (**Supplementary Figures 2A-F**). We then assessed the relationship between In-TLS density and prognosis of nmCRC patients. In-TLS was present in 26.3% of nmCRC patients and then these patients were divided into In-TLS+ and In-TLS- groups. Subsequently, in the In-TLS+ group, the receiver operating characteristics (ROC) curve for 5-year RFS was used to determine the optimum cut-off of In-TLS density and then In-TLS+ patients were divided into high-In-TLS+ (H-In-TLS+) and low-In-TLS+ (L-In-TLS+) groups. We found that the presence or density of In-TLS was not associated with prognosis (**Supplementary Figures 3A-H**).

**Table 3:** Cox proportional hazards regression models for the predictors of RFS and OS in the training set.

Variables	Univariate analyses		Multivariate analyses	
	HR (95% CI)	P value	HR (95% CI)	P value
**RFS**				
Sex (male vs female)	0.537 (0.235–1.227)	0.140		
Location (left/right colon/rectum)	1.144 (0.682–1.918)	0.610		
Age >60 vs ≤60	2.020 (0.949–4.298)	0.068		
Tumor grade (G3/G2/G1)	1.730 (0.901–3.322)	0.100		
MMR (dMMR vs pMMR)	0.420 (0.100-1.775)	0.238		
TNM stage* (III/II/I)	5.219 (2.276–11.965)	**<0.001**	4.445 (1.860–10.624)	**0.001**
Perineural invasion (no vs yes)	0.412 (0.185–0.919)	**0.030**	1.026 (0.406-2.596)	0.956
Vascular invasion (no vs yes)	0.180 (0.084–0.386)	**<0.001**	0.587 (0.249–1.384)	0.224
P-TLS density (low vs high)	6.597 (2.882–15.103)	**<0.001**	7.117 (2.478–20.437)	**<0.001**
P-TLS maturation stage (SFL/PFL/E-TLS)	0.508 (0.300–0.860)	**0.012**	1.609 (0.802–3.231)	0.181
TSP (low vs high)	0.126 (0.048–0.333)	**<0.001**	0.233 (0.080–0.679)	**0.008**
**OS**				
Sex (male vs female)	0.538 (0.236-1.230)	0.142		
Location (left/right colon/rectum)	1.137 (0.679-1.906)	0.625		
Age >60 vs ≤60	2.027 (0.953-4.315)	0.067		
Tumor grade (G3/G2/G1)	1.738 (0.902-3.348)	0.098		
MMR (dMMR vs pMMR)	0.419 (0.099-1.769)	0.236		
TNM stage* (III/II/I)	5.222 (2.278–11.975)	**<0.001**	4.276 (1.804–10.133)	**0.001**
Perineural invasion (no vs yes)	0.410 (0.184–0.914)	**0.029**	0.983 (0.370-2.380)	0.893
Vascular invasion (no vs yes)	0.173 (0.081–0.372)	**<0.001**	0.554 (0.238–1.287)	0.170
P-TLS density (low vs high)	6.628 (2.893–15.183)	**<0.001**	6.905 (2.423–19.678)	**<0.001**
P-TLS maturation stage (SFL/PFL/E-TLS)	0.505 (0.298–0.855)	**0.011**	1.529 (0.766–3.049)	0.228
TSP (low vs high)	0.125 (0.047–0.332)	**<0.001**	0.219 (0.075–0.639)	**0.005**

MMR, mismatch repair; P-TLS, peritumoral tertiary lymphoid structure; TSP, tumor stroma percentage; E-TLS, early -TLS; TSP, PFL-TLS, primary follicle-like -TLS; SFL-TLS, secondary follicle-like -TLS; PFS, progression-free survival; OS, overall survival; HR, hazard ratio; CI, confidence interval. *The 8th AJCC TNM staging system. The bold valuse was considered as statistically significant difference.

We assessed the relationship between the P-TLS maturation stages and clinical outcomes in patients. In the training set, E-TLS, PFS-TLS, and SFS-TLS were present in 22.1%, 45.1%, and 30% of nmCRC patients, respectively (**Table 1**). We found that lower P-TLS maturation stage was significantly associated with reduced RFS (HR= 0.508 95% CI: 0.300-0.860, P =0.01) and reduced OS (HR=0.505 95% CI: 0.298-0.855, P =0.011) of nmCRC patients (**Figures 4H, I**; **Table 3**). These results were validated in external validation groups (**Supplement Table 2** and **Supplementary Figures 2G, H**). However, we did not find that the In-TLS maturation stage was associated with the prognosis of nmCRC patients (**Supplementary Figures 3I-L**).

### Association of P-TLS and In-TLS in nmCRC patients

**Figure 5:**
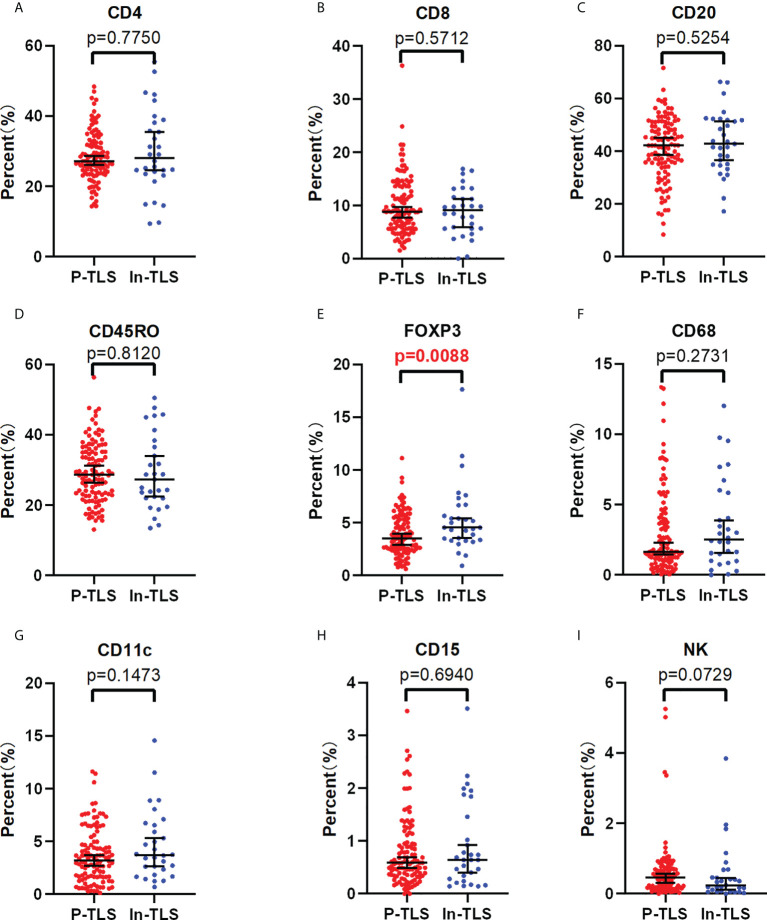
The difference between the P-TLS-cellular components and In-TLS-cellular components. **(A)** CD4+T cells; **(B)** CD8+T cells; **(C)** CD20+ B cells; **(D)** CD45RO+ lymphocytes; **(E)** FOXP3+Tregs; **(F)** CD68+TAMs; **(G)** CD11c+DC cells; **(H)** CD15+ TANs; **(I)** NCR1+NK cells.

To further explore the possible mechanism of the different prognostic values of P-TLS and In-TLS, we further evaluated the difference in the immune cell composition ratio between the two (**Figures 5A-I**). We found that In-TLS had a higher proportion of Tregs than P-TLS (**Figure 5E**). We did not find differences in the composition ratio of CD4+ T cells, CD8+ T cells, CD20+ B cells, CD45RO+ lymphocyte, CD11c+ DCs, CD68+ TAMs and CD15+ TANs between P-TLS and In-TLS.

### Characteristics of TSP and its relationship with survival of nmCRC patients

We further assessed the relationship between TSP and clinicopathological features in the training set (**Table 2**). H-TSP was observed in 47 patients (41.2%) and significantly associated with T stage (*P* = 0.0384), N stage (*P* = 0.0009), Lymph node-positive (*P* =0.0002), TNM stage (*P* =0.0009) and vascular invasion (*P* =0.0022). TSP was not significantly associated with sex, age, tumor grade and MMR status (**Table 2**). H-TSP was significantly associated with reduced RFS (HR=0.126 95% CI: 0.048-0.333, *P <*0.001, **Figure 4J**) and reduced OS (HR=0.125 95% CI: 0.047-0.332, P <0.001, **Figure 4K**) of nmCRC patients. These results were validated in external validation groups (**Supplement Table 2** and **Supplementary Figures 2I, J**).

### Association between P-TLS and TSP and its prognostic significance in nmCRC patients

We evaluated the relationship between P-TLS and TSP in the training set (**Table 2**). P-TLS density tends to be lower in H-TSP tumors (Median, 0.1130 vs 0.1710, *P* =0.0205, **Figure 3F** and **Supplementary Figure 1F**). Nevertheless, some tumors with H-TSP also exhibit high P-TLS density. Therefore, patients were divided into group1, group2 and group3 according to TSP (high and low) and P-TLS density (high and low), which were TLS^hi^TSP^lo^, TLS^hi^TSP^hi^ or TLS^lo^TSP^lo^ and TLS^lo^TSP^hi^, respectively. The TLS^hi^TSP^lo^ group achieved the best prognosis, while the TLS^lo^TSP^hi^ group showed the worst prognosis (**Figures 4L, M**; **Supplementary Figures 3K, L**). In addition, we found that TSP was not associated with the presence or density of In-TLS (**Table 2** and **Supplement Table 2**).

### Nomogram predicts the probability of 2- and 5-year RFS in nmCRC patients

**Figure 6:**
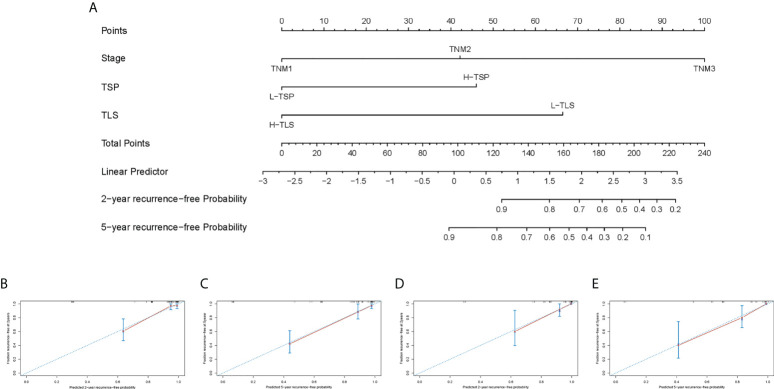
Nomogram predicts RFS and the calibration curves of the Nomogram predict RFS. **(A)** Nomogram was developed based on three factors: TNM stage, TLS and TSP to predict the probability of RFS at 2- and 5-years. The probabilities might be estimated as the sum of points for each variable as a function of total points. Each component was allocated points by drawing a line upward from the matching values to the ‘point’ line. On the “total points” line, the total sum of points added by each variable was shown. A line was drawn downward to read the associated probability forecasts. The Bootstrap method was used for internal validation, with 1000 repeat samples. Calibration curves of a nomogram to predict RFS at 2- and 5-years in the training set **(B, C)** and external validation set **(D, E)**.

Univariate Cox regression analysis showed that TNM stage, vascular invasion, perineural invasion, P-TLS density, TLS maturation stages and TSP were significantly associated with RFS and OS; and multivariate Cox regression analyses showed that TNM stage, P-TLS density and TSP were independent prognosis factors both in the training set (**Table 3**) and external validation set (**Supplement Table 3**). Based on the multivariate Cox-regression analyses, we plotted a Nomogram, including the P-TLS density, TSP and TNM stage, to predict the probability of 2- and 5-years RFS in nmCRC patients (**Figures 6A**). The Nomogram showed a superior predictive ability than the TNM stage in the training set (C-index, 0.863 vs 0.773) and external validation set (C-index, 0.857 vs 0.764). In addition, the calibration curve sets showed good agreement on actual and predicted clinical outcomes in the training set and external validation (**Figures 6B-E**).

## Discussion

By using two independent sets, we validated the role of TLS, TSP and their relationship in nmCRC patients. We characterized the spatial distribution, density and cellular components of TLS in nmCRC patients. We found that P-TLS density and TSP were significantly associated with clinical outcomes of nmCRC patients. The best survival benefit was observed in patients with high P-TLS and low P-TLS. We also found that P-TLS density was associated with TSP, and both were independent prognostic factors in nmCRC patients. Finally, we constructed a Nomogram that combined these parameters with other prognostic factors. The Nomogram performed well both internally and externally validity.

TLSs are lymphocyte-gathering structures and develop in chronic inflammatory tissues (tumors) (3). Our study showed that the presence of P-TLS was observed in 99.12% of nmCRC patients, while In-TLS was observed in 26.3%, which was roughly consistent with studies on hepatocellular carcinoma (P-TLS: 89%/95%, In-TLS: 47%/31.7%) (28, 29), lung squamous cell carcinoma (P-TLS: 97%, In-TLS: 25%) (30), and CRC studies (P-TLS: 97%) (13). We then found that high P-TLS density was associated with prolonged survival, which was broadly consistent with previous literature on CRC (13, 14). In-TLS was present in a few cases and the presence or density of In-TLS was not associated with patients’ prognosis. Similarly, Ding *et al.* (27) confirmed that P-TLS and In-TLS had opposite prognostic values in intrahepatic cholangiocarcinoma. These data could illustrate that TLSs in different locations might have different functions. In theory, In-TLS might have a more robust anti-tumor immune response than P-TLS due to its closer proximity to tumor cells, as described by Ding (27). In fact, Higher P-TLS density and maturation stage were associated with better clinical outcomes. In contrast, In-TLS density and maturation stages appeared to have no prognosis value in our cohort. Our data show that In-TLS had a higher proportion of Tregs, which may be one of the mechanisms that undermine its prognostic value. In addition, the data of Di Caro *et al.* (6) suggested that TLS density was significantly associated with the prognosis of stage II CRC patients, but not with the prognosis of stage III CRC patients. It should be noted that they defined TLS density as the percentage of TLS area to the digitized tissue surface area within three discontinuous tumor-invasive fronts (6). In this study, P-TLS density was defined as the number of TLS per square millimeter in a 7-mm area around the tumor and this predictive value was independent of TNM stage. Further prospective studies are required to confirm the performance of this method for assessing TLS density.

We also demonstrated that TLSs existed in different locations with different shapes. The mucosal TLS were usually squished and slender or teardrop-shaped. The submucosal and basal lamina propria TLS were usually oval-shaped. We further identified the cellular compositions of P-TLS. We found that most of the immune cells in P-TLS were immunocompetent, such as CD20+ B cells, CD45RO+ lymphocytes, CD4+ T cells and CD8+ T cells, but few were immunosuppressive, such as CD68+ TAMs, CD15+ TANs and FOXP3+ Treg. These findings further suggest that TLSs could support anti-tumor immune responses. Several recent studies have shown that TLSs are significantly associated with the efficacy of immune checkpoint inhibitors (ICIs). Specifically, the presence of TLSs and B cells infiltration in pretreatment biopsies in patients with melanoma, sarcoma, urothelial carcinoma and renal cell carcinoma is associated with the efficacy of ICIs (31–34). Moreover, the abundance of TLSs in melanoma correlated with PD-L1 expression on immune cells (33). The PD-1^hi^ dysfunctional CD8 T cells predominantly localized within TLS predicted response to PD-1 blockade in late-stage NSCLC (35). These findings are essential for further understanding the potential role of TLS in CRC immunotherapy.

TSP was identified in H&E-stained sections. Our data suggested that H-TSP was associated with higher T stage, lymph node metastasis, vascular invasion, higher recurrence rate and shorter survival, which is generally consistent with other studies (36, 37). Further research found that the H-TSP tumors had a lower P-TLS density. This suggests that the tumor stroma might play a key role in shaping the unfavorable microenvironment for the formation of TLSs. These data are also highlighted in the consensus molecular subtype (CMS) classification of CRC. The extensive contribution of stromal cells to the CRC mesenchymal phenotype mediates the high aggressiveness of CMS4 tumors (38, 39). In the stroma of CMS4 tumors, fibroblast, endothelial cells and monocytes express high immunosuppressive cytokines and exclude effector immune cells (40). In addition, tumor stroma would eventually mediate T cell exhaustion and immune resistance through metabolic re-editing (41). These data suggested that tumor stroma might play a detrimental role in the anti-tumor immune responses.

Although the relationship between TLS and TSP is complex, P-TLS density and TSP were independent prognostic factors of nmCRC patients. In addition, the TLS^lo^TSP^hi^ group achieved the worst prognosis, with a 5-year OS of only 25%. The formation of immunosuppressive microenvironment might be the main reason for the poor prognosis of this group of patients. Therefore, these patients would benefit from targeting and constraining the tumor stroma. Anti-stroma agents, such as transforming growth factor-beta (TGF-β), fibroblast growth factor receptors (FGFR), CXC-chemokine receptor 4 (CXCR4) and colony-stimulating factor 1 receptor (CSF1R) inhibitors are used in various preclinical and clinical development (42). For specific patients with TLS^lo^TSP^hi^, combining anti-cancer and anti-stroma agents therapy might be a promising strategy. In addition, radiation therapy has been shown to reverse tumor stroma-mediated immunosuppressive microenvironment by remodeling the tumor stroma (43). Therefore, radiotherapy combined with immunotherapy might also be a promising approach.

How to quickly and accurately identify CRC patients with poor prognoses remains a major clinical challenge. In the third-generation Memorial Sloan-Kettering Cancer Center (MSKCC) Nomogram, the addition of tumor-infiltrating lymphocytes and MMR phenotypes allowed it to more accurately predict the prognosis of colon cancer patients (44), but rectal cancer is not included in the Nomogram, which might be related to the complex treatment of rectal cancer. Although other studies have reported prediction models for CRC, these models have certain limitations, such as being based on single medical center data, and/or including only demographic information and/or clinicopathological features (45–48). Our Nomogram adds additional tumor immune microenvironment parameters, such as TLS and TSP. Although our Nomogram contains only three parameters, it has been shown to accurately predict the clinical outcomes of nmCRC patients from two independent medical centers. In addition, these parameters could be quickly obtained during routine pathological diagnosis without imposing an additional financial burden on patients.

This study also has some limitations. First, this is a retrospective study, which might have selection biases. We minimized the selection biases by rigorously adhering AJCC Precision Medicine Core checklist (49). Second, the present study cohort includes rectal and colon cancer patients without the specific location of tumors, which could achieve different postoperative survival in detail. In addition, the influence of predictive value of TLS and TSP in CRC patients receiving neoadjuvant therapy is unclear. Therefore, our follow-up study would focus on such patients, especially those with rectal cancer. Finally, although we found that the tumor stroma could shape the immunosuppressive microenvironment, it remains unclear which stromal cells or pathways are at work, and we will focus on this in the future.

## Conclusion

The present study demonstrates that P-TLS density and TSP were promising independent prognostic factors for RFS and OS in nmCRC patients. The Nomogram, including the P-TLS density, TSP and TNM stage, could more quickly and accurately predict the probability of RFS at 2- and 5-years in nmCRC patients. Further data show that H-TSP is associated with low P-TLS density. TLS and tumor stroma could provide a new therapeutic target to the tumor immune microenvironment in nmCRC patients.

## Data availability statement

The original contributions presented in the study are included in the article/**Supplementary Material**. Further inquiries can be directed to the corresponding authors.

## Ethics statement

The studies involving human participants were reviewed and approved by the Institute Research Ethics Committees of the 7th Medical Center of Chinese PLA General Hospital and Affiliated Drum Tower Hospital of Nanjing University Medical School. The patients/participants provided their written informed consent to participate in this study. Written informed consent was obtained from the individual(s) for the publication of any potentially identifiable images or data included in this article.

## Author contributions

JFD and XS came up with the concept and designed the experiments. QW and WC performed the experiments. RA and HZ provided technical support for the staining of the sections and performed standard pathologic analysis. HC, JD and WZ collected the cohort data and samples. QW and JB analyzed the data. JFD and AL contributed reagents/materials/analysis tools. QW and XS completed the manuscript, and JFD made extensive revisions to the manuscript. All authors contributed to the article and approved the submitted version.

## Funding

This work was supported by the National Natural Science Foundation of China (No. 81870393, JFD, and 81970500, XFS).

## Acknowledgments

We thank Professor Jingyun Ma for revising the language of this paper.

## Conflict of interest

The authors declare that the research was conducted in the absence of any commercial or financial relationships that could be construed as a potential conflict of interest.

## Publisher’s note

All claims expressed in this article are solely those of the authors and do not necessarily represent those of their affiliated organizations, or those of the publisher, the editors and the reviewers. Any product that may be evaluated in this article, or claim that may be made by its manufacturer, is not guaranteed or endorsed by the publisher.
