# Supplementary material for: Peritumoral tertiary lymphoid structure and tumor stroma percentage predict the prognosis of patients with non-metastatic colorectal cancer
Source: Front Immunol. 2022 Sep 16;13:962056. doi: 10.3389/fimmu.2022.962056 (PMC9524924; doi:10.3389/fimmu.2022.962056)
Supplement: Supplementary file 1 [file DataSheet_1.zip › Supplementary Material/Figure 6 data/Figure 6 B.pdf]

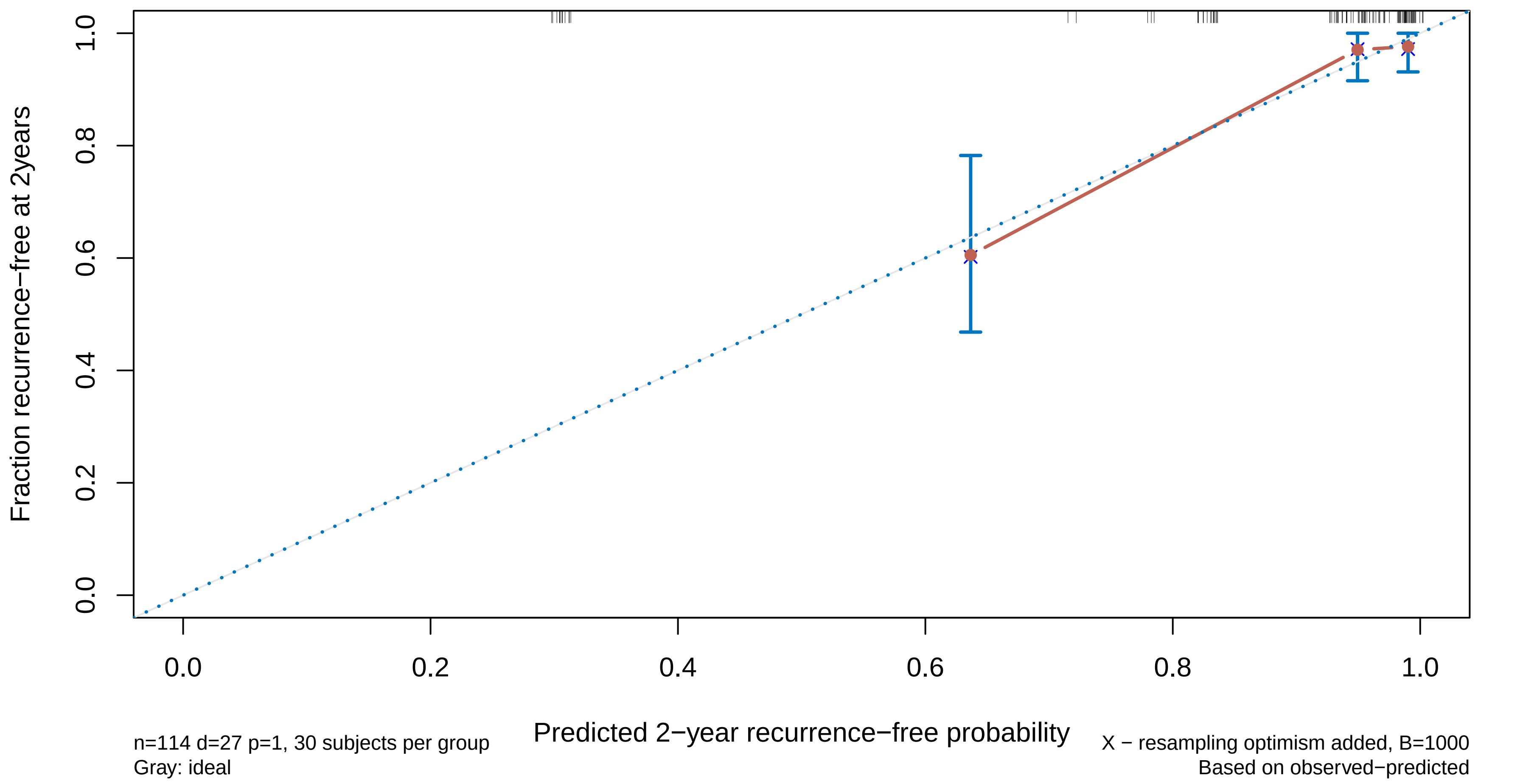

Fraction recurrence−free at 2years
0.0
0.2
0.4
0.6
0.8
1.0
Predicted 2−year recurrence−free probability
n=114 d=27 p=1, 30 subjects per group
Gray: ideal
X − resampling optimism added, B=1000
Based on observed−predicted
