# Supplementary material for: Peritumoral tertiary lymphoid structure and tumor stroma percentage predict the prognosis of patients with non-metastatic colorectal cancer
Source: Front Immunol. 2022 Sep 16;13:962056. doi: 10.3389/fimmu.2022.962056 (PMC9524924; doi:10.3389/fimmu.2022.962056)
Supplement: Supplementary file 1 [file DataSheet_1.zip › Supplementary Material/Supplementary Figures and Tables.docx]

Supplementary Material

# Supplementary Figures and Tables

##
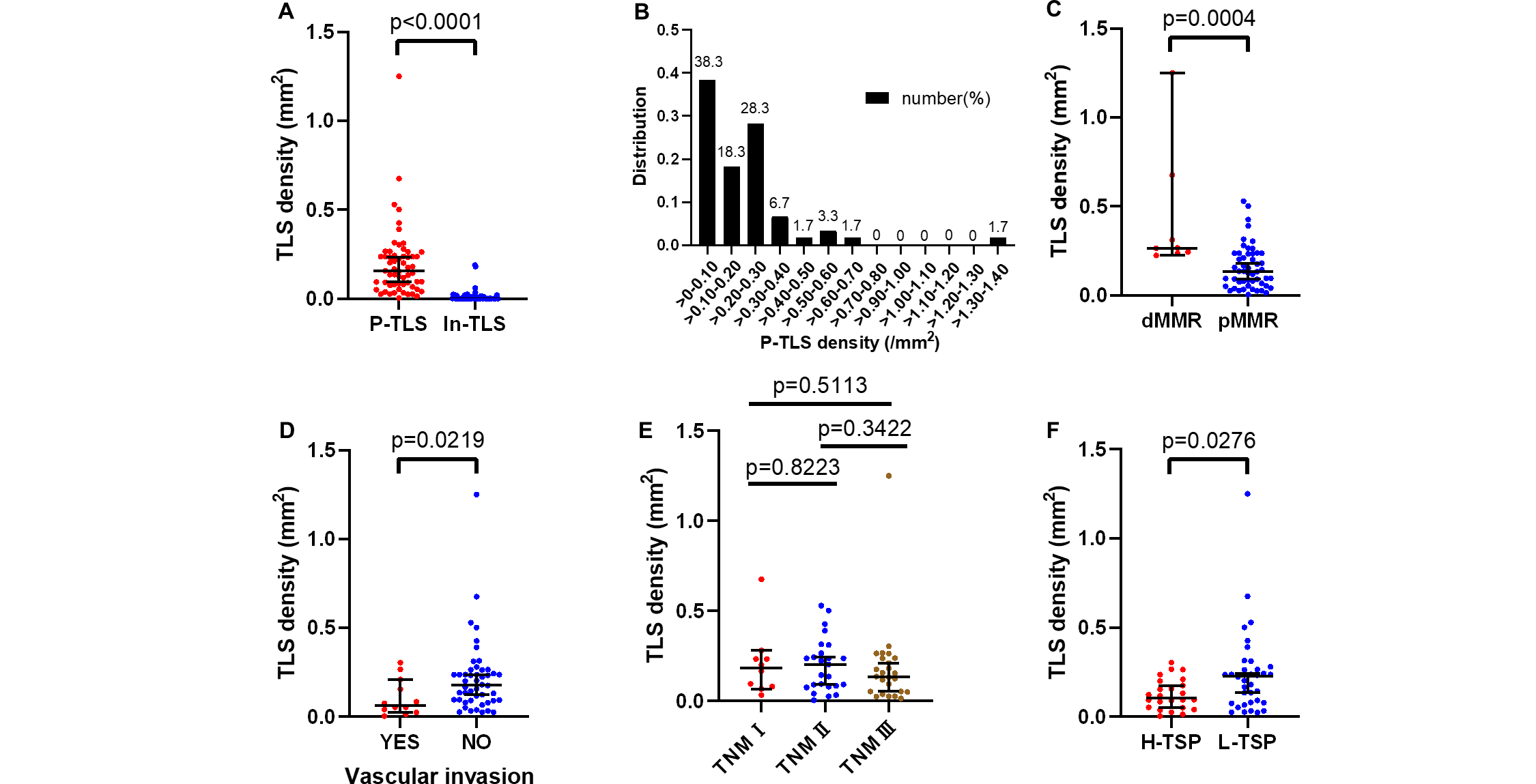
Supplementary Figures

**Supplementary Figure 1.** Distribution of P-TLS density and its relationship with clinical features in the external validation set. (A) Relationship between P-TLS density and In-TLS density; (B) istribution of P-TLS density; (C-F) Relationship between P-TLS density and MMR/Vascular invasion/TNM stage/TSP.


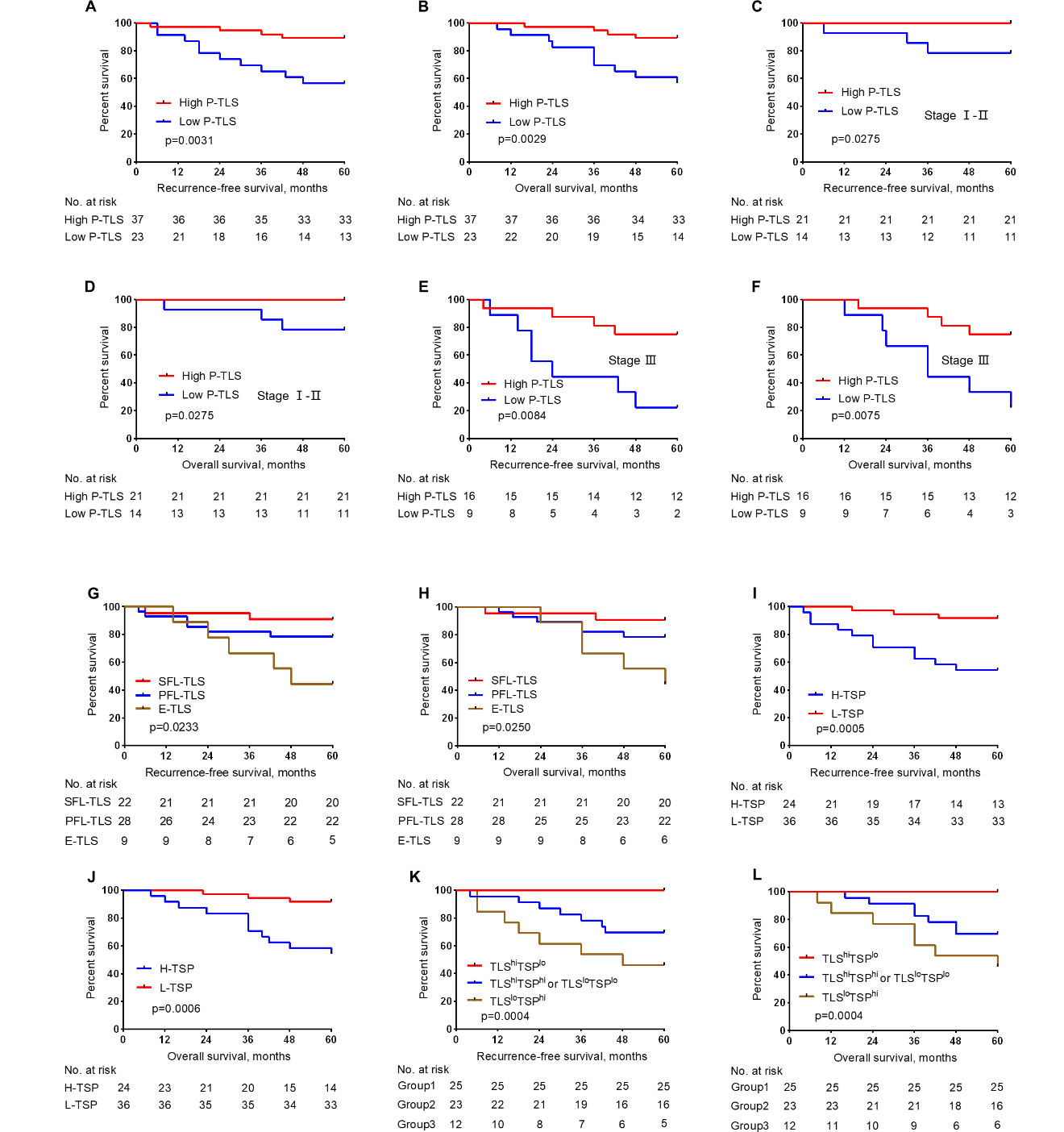
**Supplementary Figure 2.** Relationship between the P-TLS density and TSP and prognosis of nmCRC in the external validation set. Association of the P-TLS density with RFS (A) and OS (B), and TNM Ⅰ-Ⅱ (C, D) and Ⅲ (E, F) CRC patients. Association of the P-TLS maturation stage with RFS (G) and OS (H). Association of the P-TLS maturation stages with RFS (I) and OS (J) CRC patients. Association of the TSP with RFS (K) and OS (L). Kaplan-Meier survival analyses for RFS (K) and OS (L) were performed according to group 1, group 2, and group 3. Group 1: TLS^hi^TSP^lo^ group, group 2: TLS^hi^TSP^hi^ or TLS^lo^TSP^lo^ group, and group 3: the TLS^lo^TSP^hi^ group.


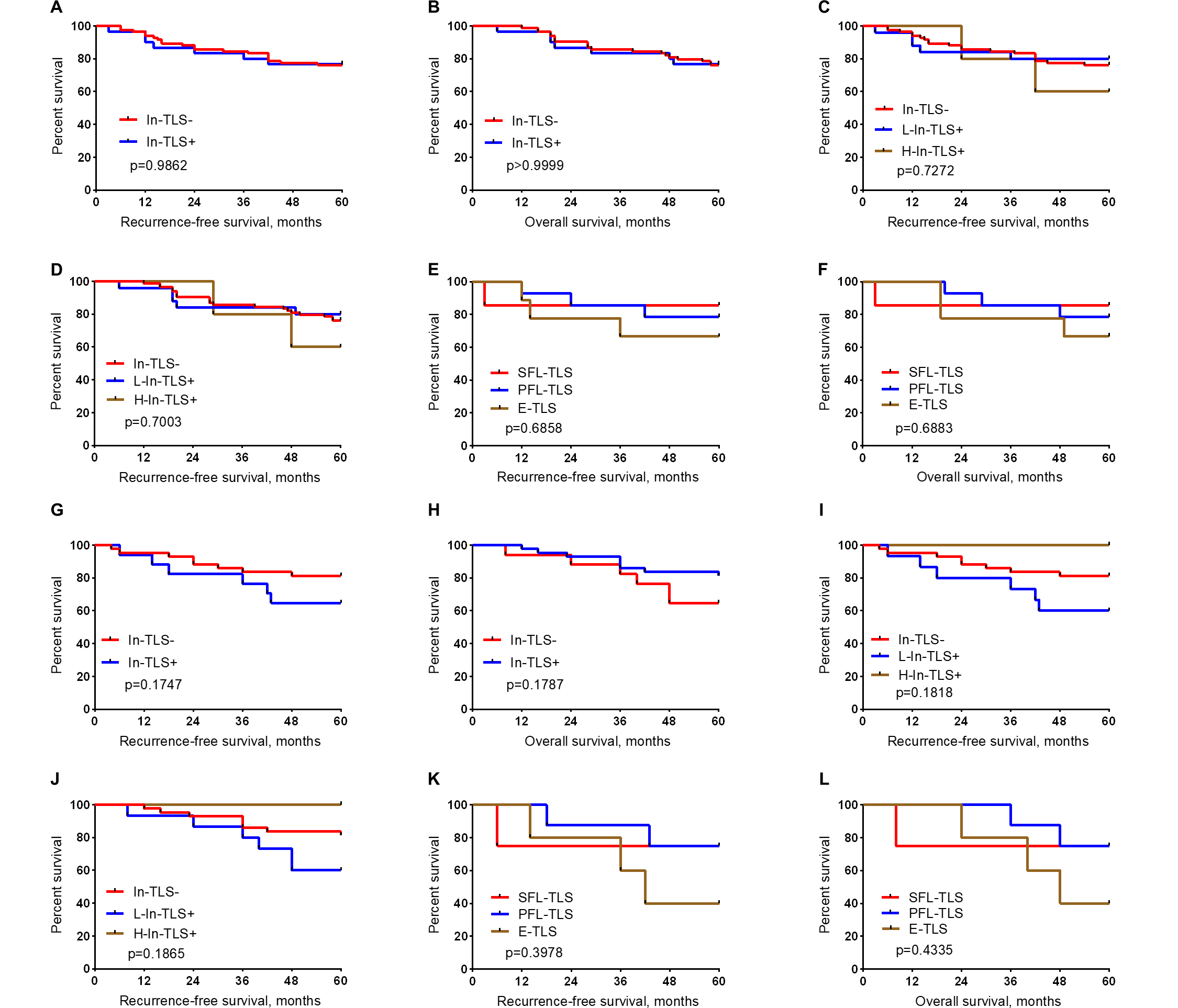


**Supplementary Figure 3.** Relationship between the In-TLS and prognosis of nmCRC. Kaplan-Meier survival analyses for RFS and OS were performed according to the presence (A, B), density (C, D) and maturation stage (E, F) of In-TLS in the training, and external validation set (G-L).

## Supplementary Tables

Supplementary Table 1 Antibodies

| **Antibody** | **Species** | **Dilution** | **Source** | **Identifier** |
| --- | --- | --- | --- | --- |
| Anti-CD3 | Mouse | 1:150 | ZSGB-BIO | TA506064 |
| Anti-CD20 | Mouse | 1:100 | Abcam | ab9475 |
| Anti-CD21 | Mouse | 1:100 | ZSGB-BIO | TA327627 |
| Anti-CD23 | Mouse | 1:150 | ZSGB-BIO | TA801554 |
| Anti-CXCL13 | Goat | 1:150 | ZSGB-BIO | ZG-0601 |
| Anti-Ki-67 | Mouse | 1:100 | ZSGB-BIO | TA800648 |
| Anti-PNAd | Rat | 1:100 | Biolegend | 120802 |
| Anti-CD4 | Rabbit | 1:200 | Abcam | ab133616 |
| Anti-CD8 | Rabbit | 1:300 | Abcam | ab101500 |
| Anti-CD11c | Rabbit | 1:500 | Abcam | ab52632 |
| Anti-CD15 | Mouse | 1:1000 | Abcam | ab17080 |
| Anti-CD45RO | Mouse | 1:1000 | Abcam | ab23 |
| Anti-CD68 | Rabbit | 1:4000 | Abcam | ab213363 |
| Anti-FOXP3 | Mouse | 1:800 | Abcam | ab20034 |
| Anti-NCR1 | Rabbit | 1:1000 | Abcam | ab224703 |
| Anti-MLH1 | Mouse | 1:150 | ZSGB-BIO | TA805066 |
| Anti-PMS2 | Mouse | 1:150 | ZSGB-BIO | TA506683 |
| Anti-MSH2 | Rabbit | 1:150 | ZSGB-BIO | ZA-0622 |
| Anti-MSH6 | Mouse | 1:150 | ZSGB-BIO | TA807929 |
| Anti-CD31 | Mouse | 1:50 | ZSGB-BIO | TA500121 |
| Anti-D2-40 | Mouse | 1:100 | ZSGB-BIO | TA327727 |
|  | | | | |

Supplementary Table 2 Relationship of TSP and P-TLS density with clinicopathological characteristics of nmCRC patients in external validation set

| **Characteristics** | **External validation set (n=60) n (%)** | | | | |
| --- | --- | --- | --- | --- | --- |
|  | **P-TLS density (median)** | **P value** | **H-TSP** | **L-TSP** | **P value** |
| **Sex** |  | 0.3275 |  |  | 0.2852 |
| Male | 0.1360 (0.0530, 0.2375) |  | 17 (70.8) | 20 (55.6) |  |
| Female | 0.2000 (0.0900, 0.2660) |  | 7 (29.2) | 16 (44.4) |  |
| **Age** |  | 0.7218 |  |  | 0.4296 |
| >60 | 0.1270 (0.0783, 0.2483) |  | 10 (41.7) | 20 (55.6) |  |
| ≤60 | 0.2015 (0.0525, 0.2435) |  | 14 (58.3) | 16 (44.4) |  |
| **Location** |  | 0.7515 |  |  | 0.9867 |
| Rectum | 0.1450 (0.0670, 0.2430) |  | 14 (58.3) | 21 (58.3) |  |
| Left colon | 0.1540 (0.0795, 0.2578) |  | 5 (20.8) | 7 (19.4) |  |
| Right colon | 0.2370 (0.0660, 0.2510) |  | 5 (20.8) | 8 (22.2) |  |
| **Lymph node-positive** |  | 0.3121 |  |  | **0.0029** |
| YES | 0.1350 (0.0520, 0.2375) |  | 16 (66.7) | 9 (25.0) |  |
| NO | 0.2000 (0.0810, 0.2660) |  | 8 (33.3) | 27 (75.0) |  |
| **Perineural invasion** |  | 0.4760 |  |  | 0.0970 |
| YES | 0.1570 (0.0270, 0.2630) |  | 7 (29.2) | 4 (11.1) |  |
| NO | 0.1540 (0.0775, 0.2405) |  | 17 (70.8) | 32 (88.9) |  |
| **Vascular invasion** |  | **0.0219** |  |  | **0.0085** |
| YES | 0.0645 (0.0283, 0.1960) |  | 9 (37.5) | 3 (8.3) |  |
| NO | 0.1780 (0.0925, 0.2580) |  | 15 (62.5) | 33 (91.7) |  |
| **T stage** |  | 0.5642 |  |  | **0.0267** |
| T1 | 0.1670 (0.0570, 0.4775) |  | 0 (0) | 5 (13.9) |  |
| T2 | 0.1760 (0.0960, 0.2330) |  | 4 (16.7) | 3 (8.3) |  |
| T3 | 0.1555 (0.0828, 0.2480) |  | 15 (62.5) | 27 (75.0) |  |
| T4 | 0.0470 (0.0230, 0.2648) |  | 5 (20.8) | 1 (2.8) |  |
| **N stage** |  | 0.5118 |  |  | **0.0053** |
| N0 | 0.2000 (0.0810, 0.2660) |  | 8 (33.3) | 27 (75.0) |  |
| N1 | 0.1445 (0.0530, 0.2233) |  | 12 (50.0) | 6 (16.7) |  |
| N2 | 0.1240 (0.0260, 0.2380) |  | 4 (16.7) | 3 (8.3) |  |
| **TNM stage*** |  | 0.5870 |  |  | **0.0057** |
| Ⅰ | 0.1835 (0.0775, 0.2448) |  | 2 (8.3) | 8 (22.2) |  |
| Ⅱ | 0.2030 (0.0815, 0.2890) |  | 6 (25.0) | 19 (52.8) |  |
| Ⅲ | 0.1350 (0.0520, 0.2375) |  | 16 (66.7) | 9 (25.0) |  |
| **Tumor grade** |  | 0.6099 |  |  | 0.2456 |
| G1 | 0.2000 (0.1240, 0.2660) |  | 2 (8.3) | 9 (25.0) |  |
| G2 | 0.1540 (0.0670, 0.2430) |  | 17 (70.8) | 22 (61.1) |  |
| G3 | 0.1060 (0.0465, 0.2438) |  | 5 (20.8) | 5 (13.9) |  |
| **MMR** |  | **0.0004** |  |  | 0.4571 |
| dMMR | 0.2655 (0.2385, 0.5843) |  | 2 (8.3) | 6 (16.7) |  |
| pMMR | 0.1335 (0.0565, 0.2360) |  | 22 (91.7) | 30 (83.3) |  |
| **TSP** |  | **0.0276** | - | - | - |
| H-TSP | 0.1070 (0.0515, 0.1940) |  | - | - |  |
| L-TSP | 0.2285 (0.0795, 0.2765) |  | - | - |  |
| **Presence of In-TLS** |  | - |  |  | 0.5640 |
| Yes | - |  | 8 (33.3) | 9 (25.0) |  |
| No | - |  | 16 (66.7) | 27 (75.0) |  |
| **In-TLS density** | - | - | 0.0075 | 0.0141 | 0.6445 |
| P-TLS, peritumoral tertiary lymphoid structure; In-TLS, intratumoral TLS; TSP, tumor stroma percentage; H-TSP, high TSP; L-TSP, low TSP; nmCRC, non-metastatic colorectal cancer; MMR, mismatch repair. *The 8th AJCC TNM staging system | | | | | |

Supplementary Table 3 Cox proportional hazards regression models for the predictors of PFS and OS in the External validation set

| **Variables** |  | **Univariate analyses** | |  | **Multivariate analyses** | |
| --- | --- | --- | --- | --- | --- | --- |
|  |  | **HR (95% CI)** | **P value** |  | **HR (95% CI)** | **P value** |
| **RFS** |  |  |  |  |  |  |
| Sex (male vs female) |  | 0.917 (0.307–2.738) | 0.877 |  |  |  |
| Location (left/right/rectum) |  | 1.209 (0.599–2.439) | 0.596 |  |  |  |
| Age >60 vs ≤60 |  | 0.356 (0.112–1.137) | 0.081 |  |  |  |
| Tumor grade (G3/G2/G1) |  | 2.206 (0.901–5.403) | 0.083 |  |  |  |
| MMR (dMMR vs pMMR) |  | 0.448 (0.059–3.423) | 0.439 |  |  |  |
| TNM stage* (III/II/I) |  | 5.185 (1.598–16.820) | **0.006** |  | 9.541 (1.598–56.950) | **0.013** |
| Perineural invasion (no vs yes) |  | 0.240 (0.083–0.694) | **0.008** |  | 0.462 (0.143–1.498) | 0.198 |
| Vascular invasion (no vs yes) |  | 0.161 (0.056–0.469) | **0.001** |  | 0.561 (0.173–1.822) | 0.336 |
| P-TLS density (low vs high) |  | 4.851 (1.518–15.502) | **0.008** |  | 8.996 (1.555–52.043) | **0.014** |
| P-TLS maturation stage (SFL/PFL/E-TLS) |  | 0.373 (0.169–0.820) | **0.014** |  | 1.552 (0.491–4.910) | 0.454 |
| TSP  (low vs high) |  | 0.143 (0.040–0.515) | **0.003** |  | 0.211 (0.052–0.857) | **0.030** |
| **OS** |  |  |  |  |  |  |
| Sex (male vs female) |  | 0.924 (0.310–2.758) | 0.887 |  |  |  |
| Location (left/right/rectum) |  | 1.197 (0.594–2.413) | 0.615 |  |  |  |
| Age >60 vs ≤60 |  | 0.361 (0.113–1.152) | 0.085 |  |  |  |
| Tumor grade (G3/G2/G1) |  | 2.286 (0.925–5.650) | 0.073 |  |  |  |
| MMR (dMMR vs pMMR) |  | 0.445 (0.058–3.400) | 0.435 |  |  |  |
| TNM stage* (III/II/I) |  | 5.085 (1.570–16.467) | **0.007** |  | 8.834 (1.507–51.780) | **0.016** |
| Perineural invasion (no vs. yes) |  | 0.243 (0.084–0.705) | **0.009** |  | 0.458 (0.142–1.479) | 0.192 |
| Vascular invasion (no vs yes) |  | 0.161 (0.056–0.467) | **0.001** |  | 0.492 (0.154–1.575) | 0.232 |
| P-TLS density (low vs high) |  | 4.864 (1.523–15.534) | **0.008** |  | 8.540 (1.522–47.909) | **0.015** |
| P-TLS maturation stage (SFL/PFL/E-TLS) |  | 0.377 (0.171–0.831) | **0.016** |  | 1.589 (0.494–5.116) | 0.437 |
| TSP  (low vs high) |  | 0.147 (0.041–0.528) | **0.003** |  | 0.236 (0.059–0.945) | **0.041** |
| MMR, mismatch repair; P-TLS, peritumoral tertiary lymphoid structure; TSP, tumor stroma percentage; E-TLS, early -TLS; TSP, PFL-TLS, primary follicle-like -TLS; SFL-TLS, secondary follicle-like -TLS; PFS, progression-free survival; OS, overall survival; HR, hazard ratio; CI, confidence interval. *The 8th AJCC TNM staging system | | | | | | |
