# Supplementary figures and images for: Peritumoral tertiary lymphoid structure and tumor stroma percentage predict the prognosis of patients with non-metastatic colorectal cancer
Source: Front Immunol. 2022 Sep 16;13:962056. doi: 10.3389/fimmu.2022.962056 (PMC9524924; doi:10.3389/fimmu.2022.962056)

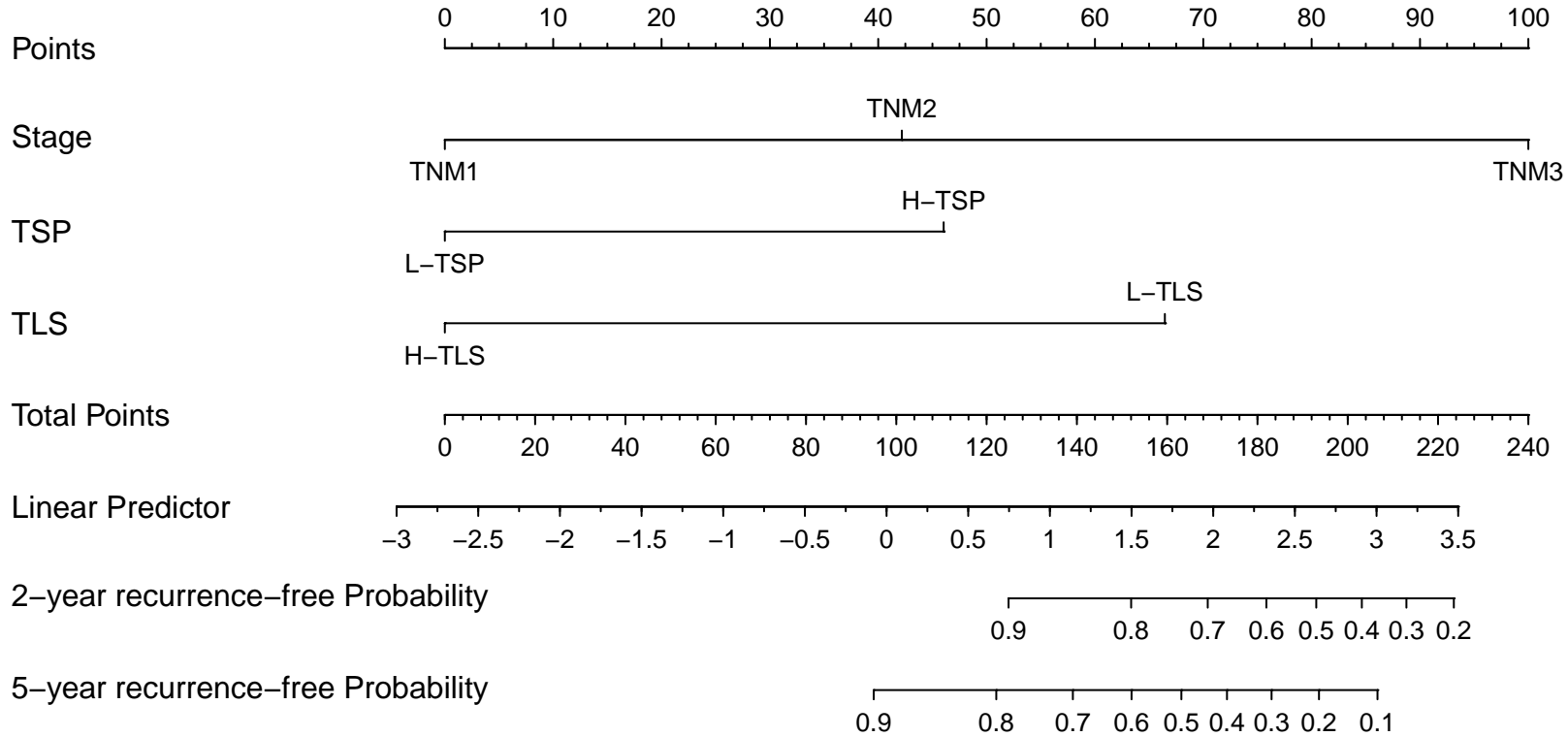

Supplement: Supplementary file 1 [file DataSheet_1.zip › Supplementary Material/Figure 6 data/Figure 6 A-1.pdf]

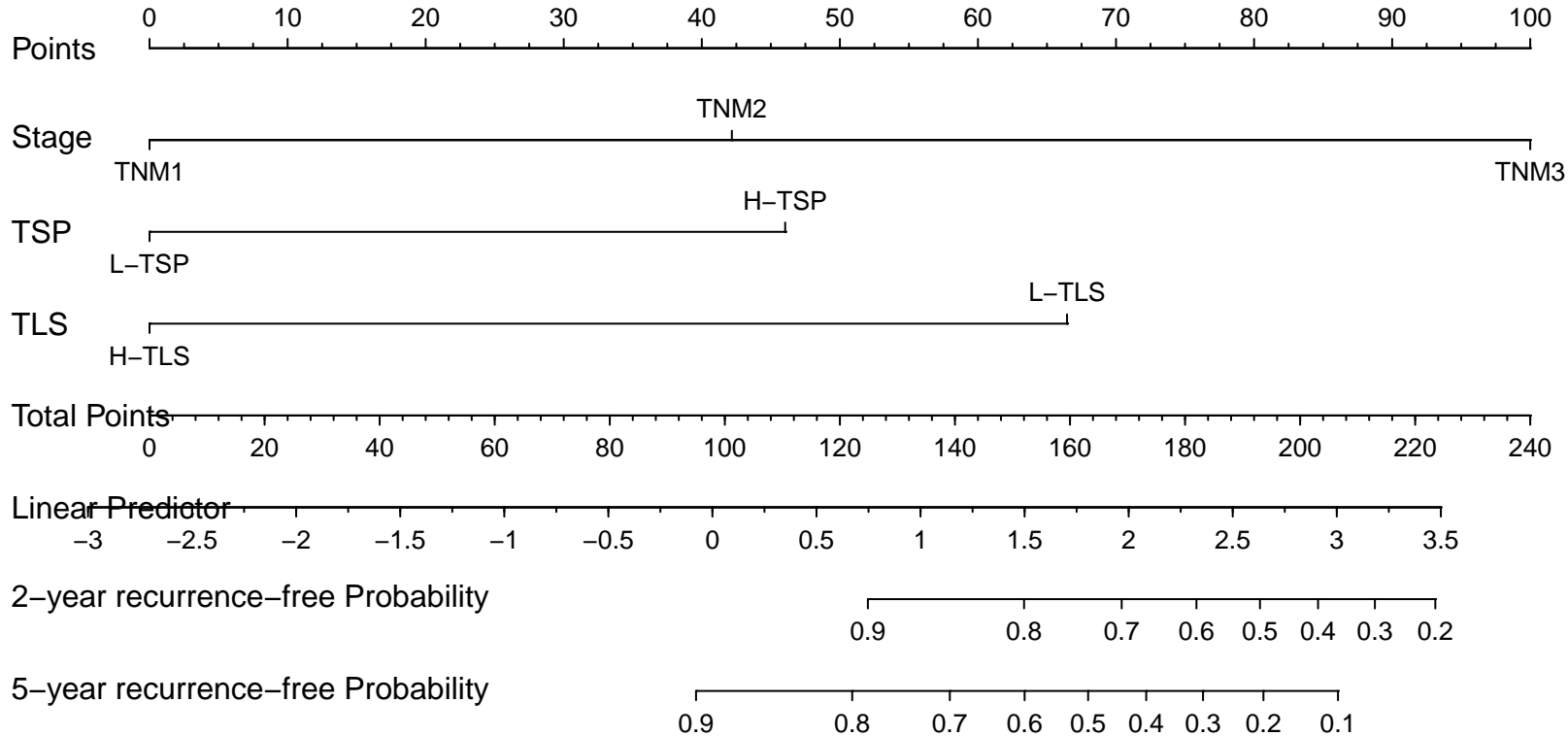

Supplement: Supplementary file 1 [file DataSheet_1.zip › Supplementary Material/Figure 6 data/Figure 6 A-2.pdf]

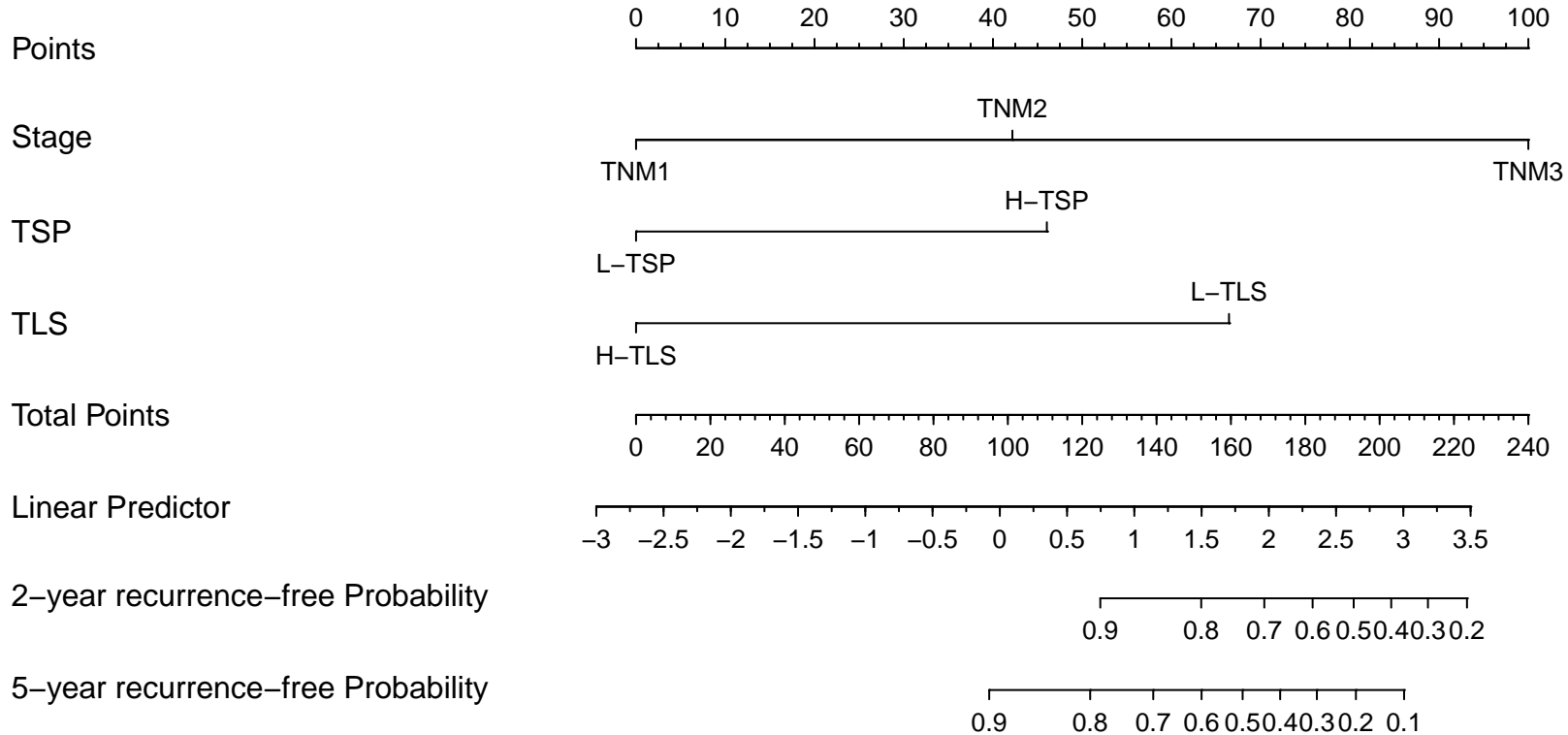

Supplement: Supplementary file 1 [file DataSheet_1.zip › Supplementary Material/Figure 6 data/Figure 6 A-3.pdf]

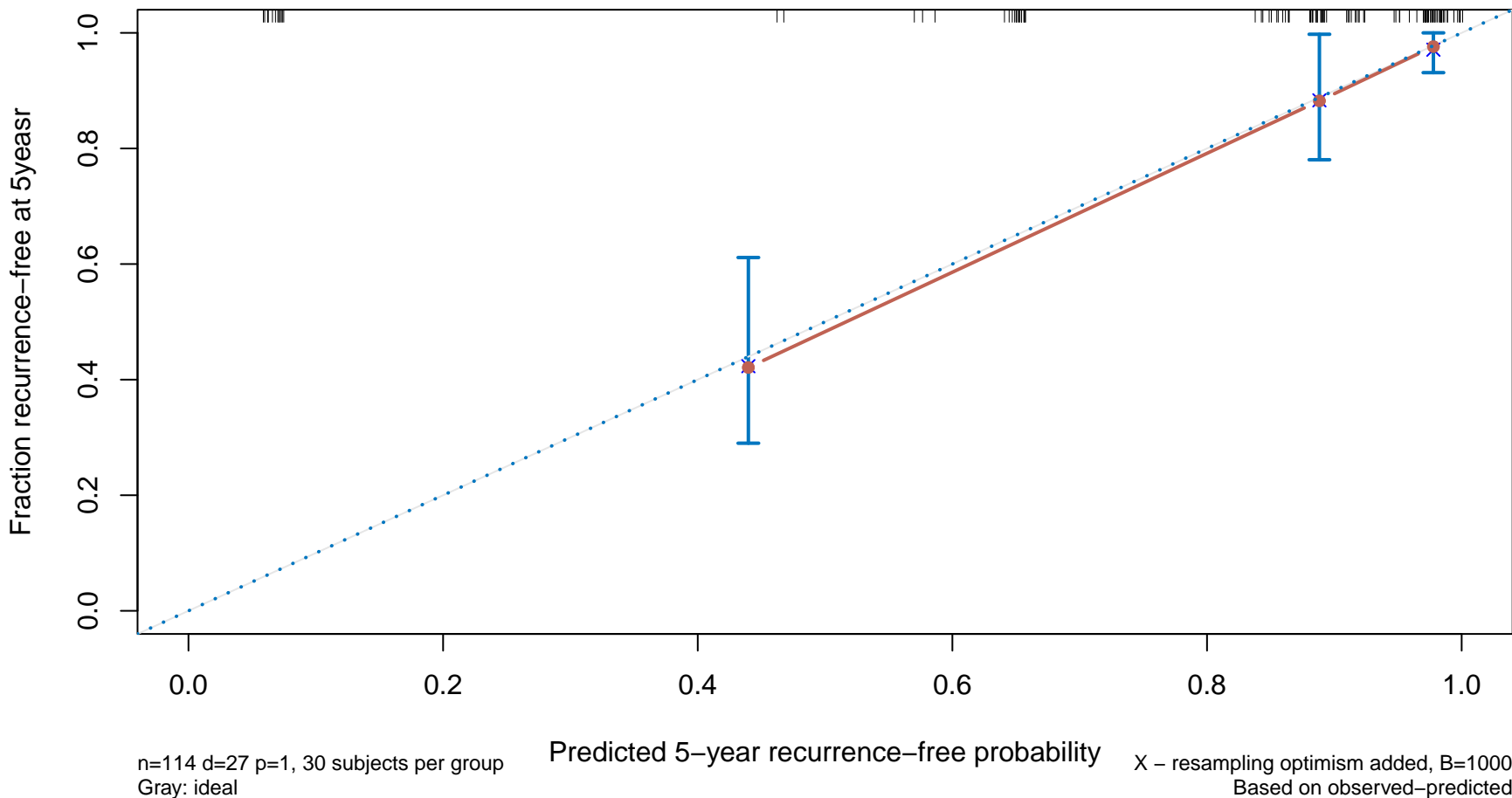

Supplement: Supplementary file 1 [file DataSheet_1.zip › Supplementary Material/Figure 6 data/Figure 6 C.pdf]

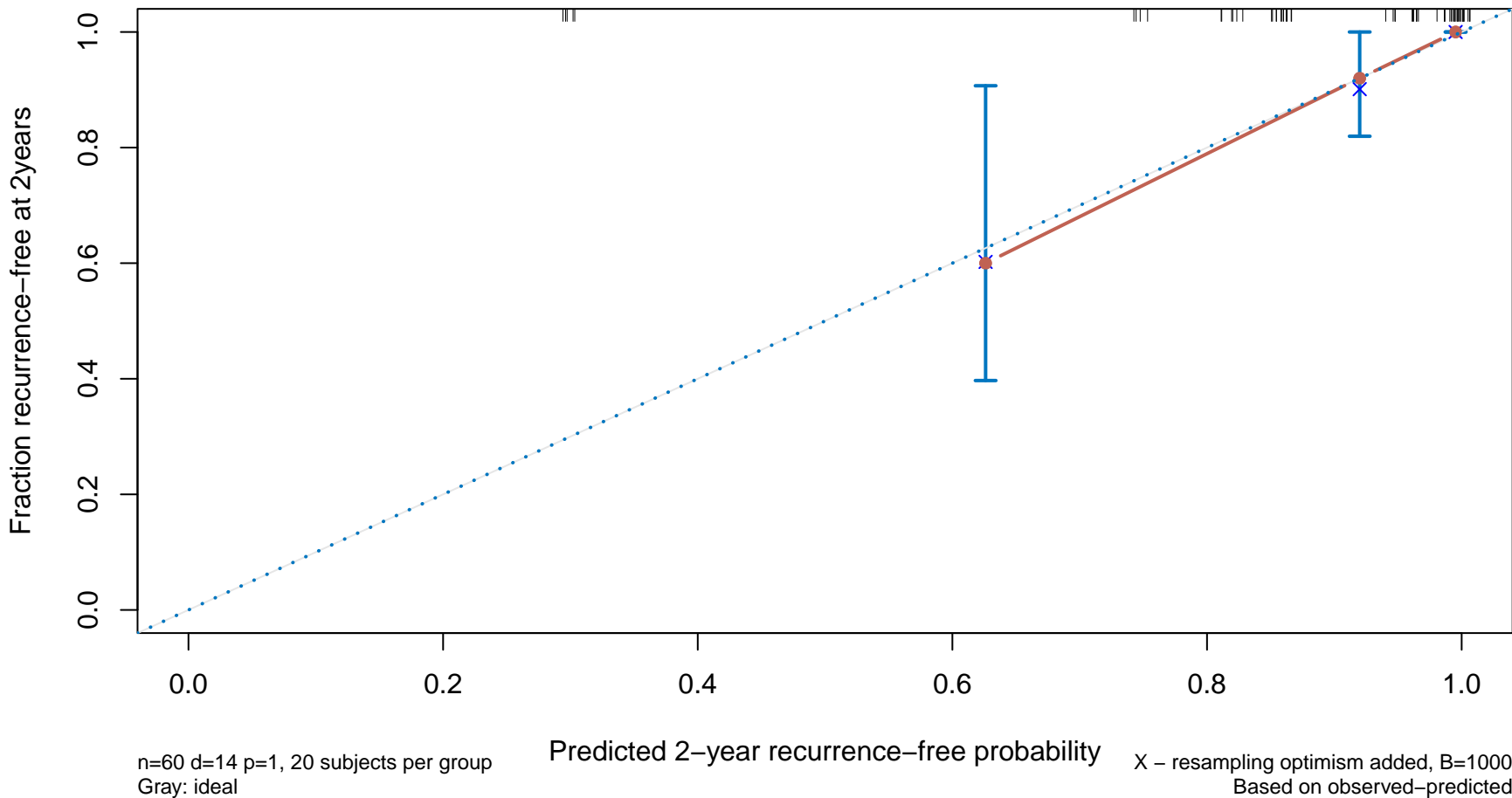

Supplement: Supplementary file 1 [file DataSheet_1.zip › Supplementary Material/Figure 6 data/Figure 6 D.pdf]
